# Statins reduce endogenous dolichol levels in the neuroblastoma cell line SH-SY5Y

**DOI:** 10.1186/2050-6511-13-S1-A51

**Published:** 2012-09-17

**Authors:** Bihter Atil, Evelyn Sieczkowski, Martin Hohenegger

**Affiliations:** 1 Institute of Pharmacology, Center for Physiology and Pharmacology, Medical University Vienna, 1090 Vienna, Austria

## Background

Statins are derived from fungi metabolites and are able to inhibit HMG-CoA reductase in the mevalonate pathway. Based on this feature, statins are safely and successfully used in the treatment of cardiovascular diseases correlated with hypercholesterolemia. Additionally, several studies have shown that statins have also pleiotropic effects, like anti-inflammatory, anti-thrombogenic, and anti-proliferative actions. Previously, we were able to show that statins have the potential to directly inhibit the ATP-binding cassette transporter B1 (ABCB1) which plays a key role in the chemoresistance of several tumor types [1].

## Methods

In add-back assays the simvastatin-induced caspase 3 activation was measured using fluorescent caspase 3 substrate. Similarly, glycosylation of ABCB1 was determined by Western blot analysis. The endogenous dolichol levels were quantified by thin layer chromatography (TLC) and high performance liquid chromatography (HPLC) in SH-SY5Y cells.

## Results

Simvastatin reduced the amount of the mature-glycosylated form (180 kDa) while increasing the core-glycosylated form (140 kDa) of ABCB1. However, this effect and the apoptosis induction by simvastatin were reversible by addition of dolichol in a time- and concentration-dependent manner. Furthermore, our HPLC analyses proved that endogenous dolichol levels were significantly decreased by simvastatin treatment for 48 hours. Effects of simvastatin concentrations as low as 0.1 µM were significant. Finally, activation of caspase 3 triggered by simvastatin was prevented by addition of dolichol.

## Conclusions

Here we show that simvastatin is able to reduce dolichol levels in SH-SY5Y cells. The endogenous dolichol depletion caused by simvastatin might influence glycosylation process in the ER leading to alterations of glycosylation pattern of ABCB1. Moreover, our data suggest that this correlation between simvastatin and decreased endogenous dolichol levels also correlates with the apoptotic potential of simvastatin.
